# Actionable health app evaluation: translating expert frameworks into objective metrics

**DOI:** 10.1038/s41746-020-00312-4

**Published:** 2020-07-30

**Authors:** Sarah Lagan, Patrick Aquino, Margaret R. Emerson, Karen Fortuna, Robert Walker, John Torous

**Affiliations:** 1 Department of Psychiatry, Beth Israel Deaconess Medical Center, Harvard Medical School, Boston, MA 02115 USA; 2 Department of Psychiatry, Lahey Hospital and Medical Center, Boston, MA 01805 USA; 3 College of Nursing, University of Nebraska Medical Center, Omaha, NE 68198 USA; 4 Department of Psychiatry, Geisel School of Medicine, Dartmouth College, Hanover, NH 03755 USA; 5 Department of Mental Health, Office of Recovery and Empowerment, Boston, MA 02114 USA

**Keywords:** Adverse effects, Translational research

## Abstract

As use and availability of mobile health apps have increased, so too has the need for a thorough, accessible framework for app evaluation. The American Psychiatric Association’s app evaluation model has emerged as a way to critically assess an app by considering accessibility, privacy and security, clinical foundation, engagement, and interoperability; however, there is no centralized database where users can view how various health apps perform when assessed via the APA model. In this perspective, we propose and outline our effort to translate the APA’s model for the evaluation of health apps into a set of objective metrics that can be published online, making the framework actionable and accessible to a broad audience. The questions from the APA model were operationalized into 105 objective questions that are either binary or numeric. These questions serve as the foundation of an online database, where app evaluation consists of answering these 105 questions and can be crowdsourced. While the database has yet to be published and crowdsourced, initial internal testing demonstrated excellent interrater reliability. The database proposed here introduces a public and interactive approach to data collection that is guided by the APA model. The published product enables users to sort through the many mobile health apps and filter them according to individual preferences and priorities, making the ever-growing health app market more navigable.

## The need for a comprehensive app evaluation framework

The need for accessible mental healthcare is more urgent than ever. For example, in 2016, mental health conditions impacted more than a billion people worldwide and depression in 2020 is recognized by the World Health Organization as a leading global cause of disability^1^. Despite efforts to improve access, significant disparities in access to mental healthcare persist in every country in the world. In recent years, digital health interventions such as smartphone apps have emerged as potentially cost-effective, evidence-based, and scalable tools to expand access to mental healthcare worldwide. The proliferation of healthcare apps, potentiated by expanding smartphone ownership and internet connectivity^2^, has been rapid: there are already an estimated 350,000 health apps with 10,000 focused on mental health^3^. Yet, despite the vast numbers of mobile apps available, the adoption of these tools is variable, with associated challenges within the context of standardization, provider, and patient levels.

The marketplace of mental health apps continues to grow and change at a rapid pace, prompting questions about how to assess quality and effectiveness. Given the dynamic nature of the digital health app space, it is difficult for service users, peer support specialists, and clinical providers alike to stay updated and ensure that apps are safe, evidence based, usable, and clinically meaningful. As an example of the challenge, a clinically relevant app for depression becomes unavailable and deleted from the app stores every 2.9 days^4^. Providers seeking to utilize apps to support patient management have reservations in recommending apps as a treatment given the limited oversight and accountability that exists with any one app^5^. Complicating matters further, for the general public today, healthcare providers are not the main source of information regarding health apps—individuals are more likely to rely upon app store reviews and rankings to decide on an app for health^6^. However, these app store rankings are marketing metrics not aligned with clinical guidelines or utility^7^. There are mounting concerns about quality and safety even among top-ranked apps in the commercial marketplaces^8^.

Despite broad regulatory efforts in the digital health space, health apps have largely escaped oversight. The US Food and Drug Administration (FDA) released a set of guidelines for regulating mobile medical apps in 2015^9^. The guidelines impose a thorough set of standards, including those for labeling, medical claims, safety, and effectiveness. Because most apps are categorized as “health and wellness” apps, however, they are not designated as medical devices and thus fall outside the purview of these FDA guidelines. Those which may be medical apps have utilized the regulatory discretion pathway to avoid scrutiny. The app stores, which have emerged as the major sources of information in the absence of FDA assessment, are ill-equipped to provide the thorough expert analysis of accreditation in their current format of user rankings and reviews.

Various app ranking models have emerged to fill this void and provide a source of clarity and objectivity in app evaluation. Although there are now upwards of forty-five different frameworks for the evaluation of mobile apps, none of the existing frameworks are suitable for use in health technology assessment (HTA) to inform policymakers, individuals, and providers because they neglect to evaluate both the potential for harm and the effect of software updates^10^. Many of these ranking systems rely upon expert consensus, which can be opaque and difficult to understand for both users and clinicians. Furthermore, there is still significant inconsistency in their outcomes. For example, a study of three different ranking systems (Psyberguide, ORCHA, MindTools.io) demonstrated a lack of correspondence in evaluating top apps, with Fleiss’ Exact Kappa scores for three domains ranging from 0.147 (for data use and security) to 0.228 (for credibility and evidence base), indicating weak reliability^11^. As a potential solution, the FDA has amended its effort towards evaluation of mobile health apps, adopting a “Pre-Certification” model that will focus efforts on app developers more than the evaluation of individual apps themselves^12^. While the FDA’s Pre-cert initiative holds promise, it is already the topic of political debate and proving its utility, as well as engaging developers may prove to be a slow process. In the meantime, there is a necessity for a framework tailored to clinicians and individuals’ needs today as they determine what apps suit their needs.

We sought to develop a framework for the assessment of health apps that would augment available evaluation models and help individuals harness the potential of digital health by choosing a relevant, safe, and effective app. This model was developed in collaboration with the American Psychiatric Association’s (APA) app evaluation framework^13^ and builds off the original model, published in June 2019, and endorsed by the APA in 2017. As the first app evaluation model to be endorsed by a major medical society, the framework reflects consensus from diverse stakeholders including service users, social workers, psychiatrists, psychologists, trainees, and informaticists. However, despite the name there is nothing specific to mental health about the model or its contents; the process of evaluation is suitable for any type of mobile health app. The APA app evaluation model is already well accepted and has been used by the New York Department of Health in the construction of an app library suited to local needs^14^.

The framework was constructed via a six-step process that involved harmonizing the 961 questions from 45 existing app evaluation frameworks, removing redundant questions, and grouping the remaining 357 into five priority levels: background info, privacy and safety, evidence, ease of use, and data integration^15^. The framework proposed here is similar in form and content to the initial APA model, with the five levels arranged in a pyramid format to reinforce the need to consider access, safety, and privacy first. There are some additions and alterations to several questions to reflect ongoing feedback from stakeholders after a two-day summit in December of 2019 (Supplementary Note 1).

### From framework to platform: development of a database

While the APA model provides a useful model through which to consider health apps and make informed decisions, it may be overwhelming for a single clinician during a short clinical visit to attempt to rigorously analyze the many apps that may be relevant to an individual with a particular condition and preferences. To make this framework functional and actionable for the public use, we adapted the questions for inclusion in a database. Each question was operationalized so that answers are binary or numeric, permitting objectivity. This resulted in 105 questions. In contrast to many existing frameworks and rating systems, many of which rely upon subjective quality and perceived impact, the assessment of an app is intended to be data-driven rather than derived from ratings of expert consensus. That said, our model is complementary and compatible with many other impressive app evaluation efforts as the 105 questions we ask of an app are often reflected in other frameworks, including the widely used Mobile App Rating (MARS) scale^16^ and mHIMSS framework^17^, as well as the more recently developed Standards for Mobile Health-Related Apps^18^. The main difference is that we do not score questions or produce summary scores, but instead let the end user judge what is important and a good match for then. Ultimately, we designed the model to be self-sustaining and fully functional for use by a single clinician or patients.

An additional benefit of the 105 objective questions is the opportunity for crowdsourcing. Since there is no qualitative assessment involved, there is great potential to involve many people in the evaluation process and offer clear quality controls. This crowdsourcing is an integral component of maintaining an up-to-date and thorough database that reflects the wide-reaching, fast-moving nature of the mental health app space. In order for rapid knowledge synthesis to be obtained from crowdsourcing, the information needs to be accessible, cost-effective, and scalable. Creating such a crowdsourced model offers the advantage of involving all stakeholders, encouraging diversity, and quickly identifying unsafe apps as outlined in our group’s recent proposed around regulating digital health technologies with transparency^19^.

In creating questions for this new database, we sought to align closely with the APA pyramid framework’s key questions, but there are several key differences. Although there are questions pertaining to each level of the pyramid (access/functionality, privacy, evidence, usability, interoperability), additional questions were added by a team of researchers to highlight further data that can be objectively coded about apps including data input methods, app outputs, and engagement styles offered. These questions were derived from prior research examining how attributes of top-rated apps relate to quality^20^ and refined through consensus in rating over 100 apps with them. Further feedback was sought from end users and clinicians to refine the clarify and focus of these questions. Consensus was obtained from twenty individuals who rated at least two apps and participated in focus groups to offer feedback on the process. While answering 105 questions about an app is of course not a rapid process, the end product of an easily searchable and updatable database enabling users to immediately sort apps according to the presence or absence of different features relevant to each unique clinical case is appealing. As with the APA model, there is no single score assigned to an app; rather, the database enables customization in consideration of various app aspects.

## A pyramid process: components of the framework

**Fig. 1:**
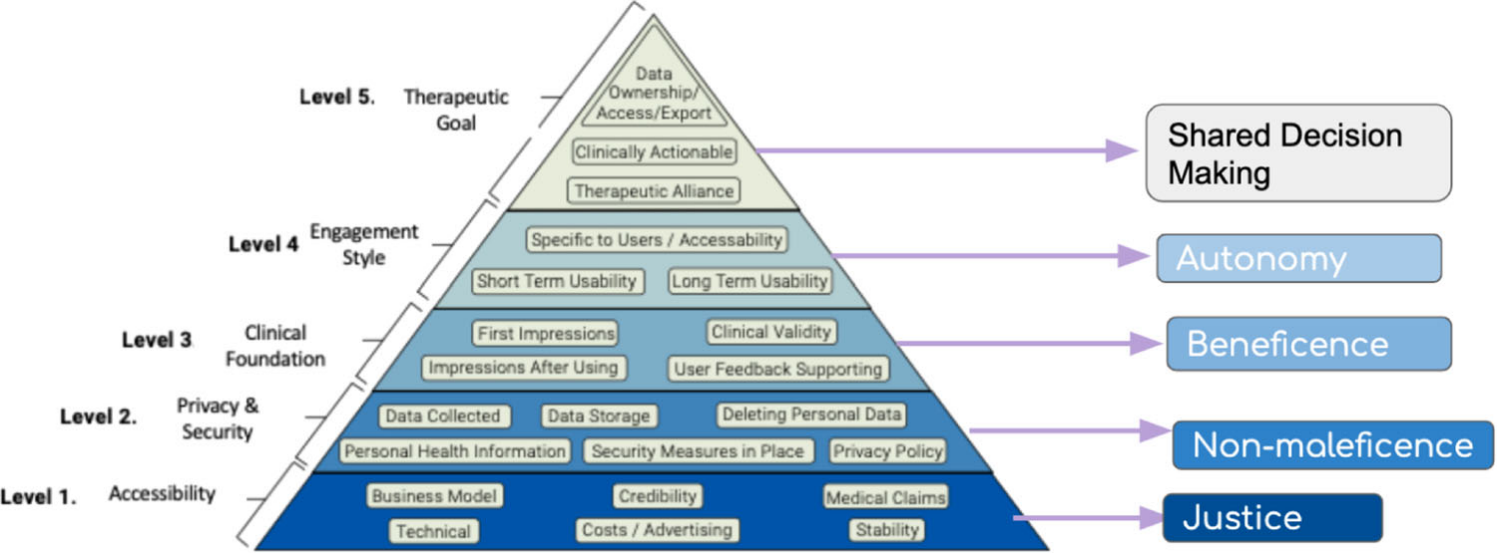
APA Framework. The pyramid depicts the APA Framework and the ethical principle corresponding to each level.

The five levels of the APA framework are: (1) Background and access, (2) Data safety and privacy, (3) App effectiveness and clinical foundation, (4) User engagement, (5) Data integration towards therapeutic alliance (Fig. 1). Associated with each level is a series of questions intended to facilitate dialogue between a clinician and an individual that will lead to the choice of the most therapeutically valuable app (Appendix A). The pyramid shape is to encourage users to start at the bottom and work their way up: if the app is unable to provide the data security that an individual seeks, for example, the evaluation need not continue up the rest of the pyramid. Each level corresponds to a principle of medical ethics, grounding the framework in enduring values that compose the overarching skeleton even as individual questions may be altered or added. To develop the framework, each of the original APA questions was operationalized such that it could be answered objectively (with either a binary or numeric response). The progression from APA framework level to database question is depicted in Supplementary Table 1.

### Background and access

**Fig. 2:**
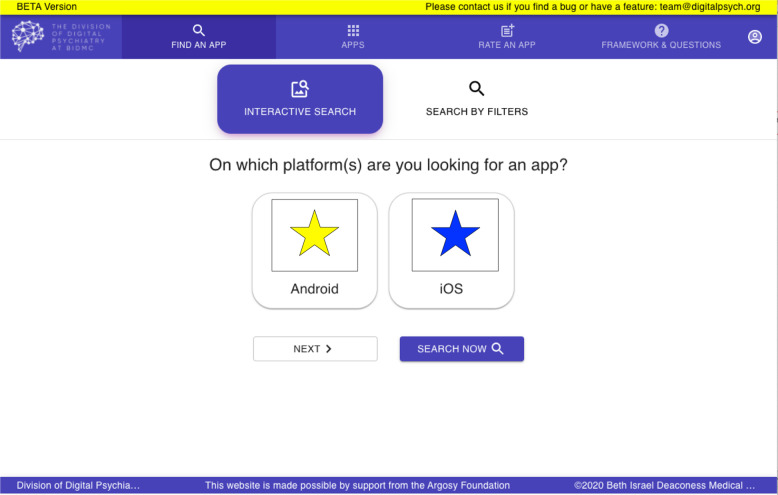
Database Landing Page. The first page that greets users is an interactive search for an app.

**Fig. 3:**
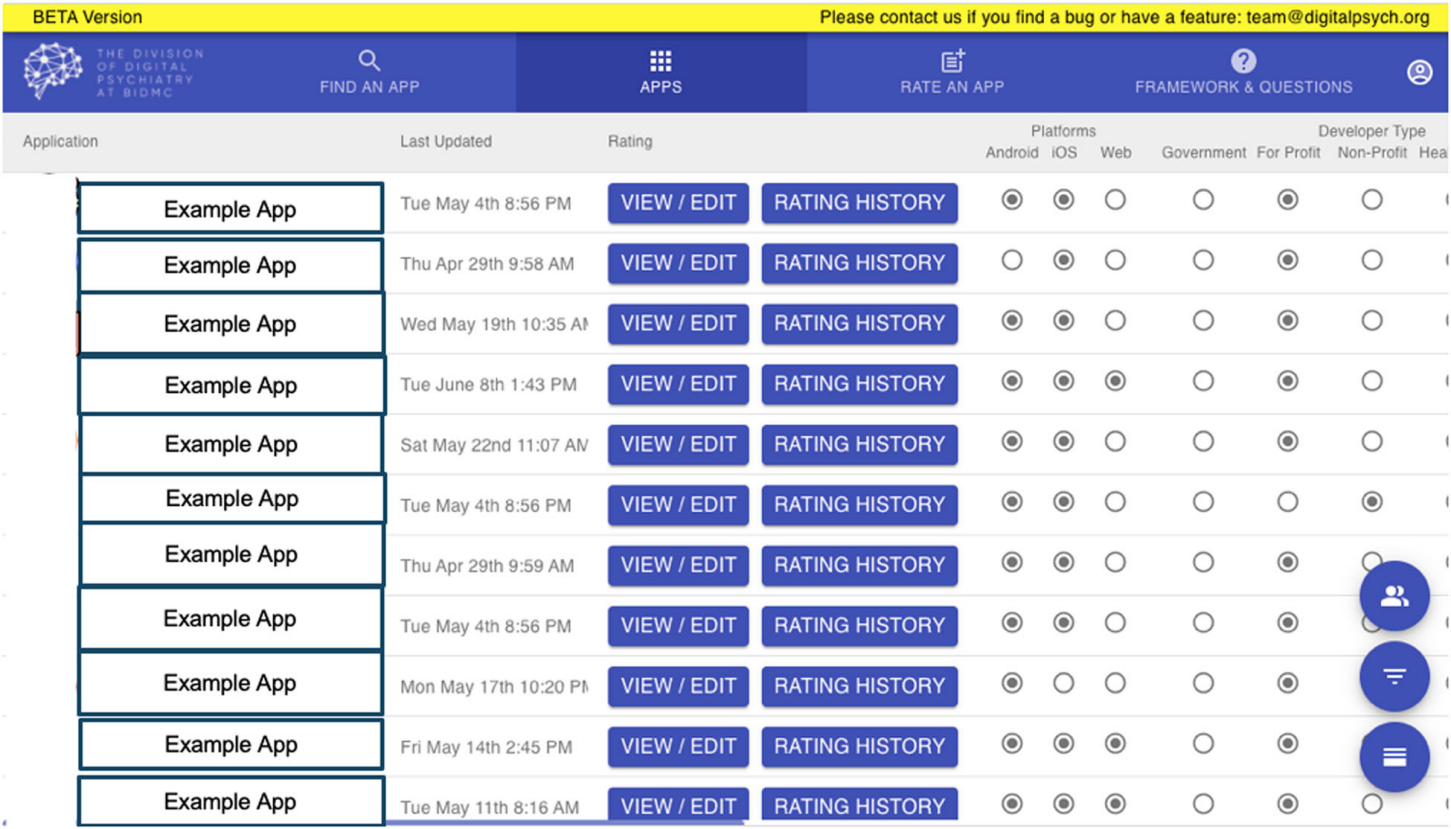
Database Sort App Feature. Users can sort apps based on desired criteria and compare features.

**Fig. 4:**
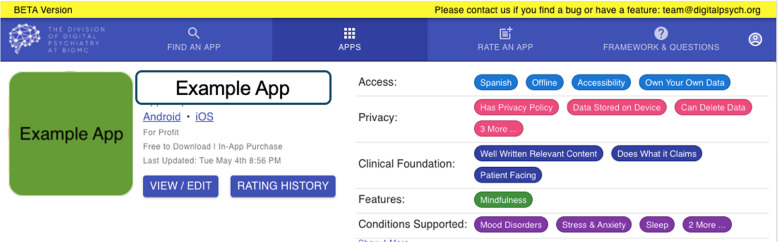
Database List View. The app attributes are clearly depicted in list view, with users able to view app attributes across the APA categories.

**Fig. 5:**
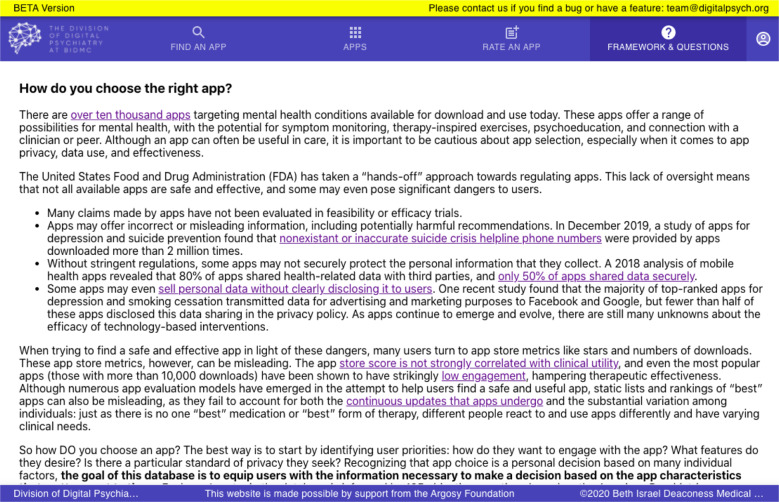
Database Informational Modules. The database offers informational modules in conjunction with the ability to find and filter apps.

Grounded in the ethical principle of justice, this level is concerned with ensuring the benefits of apps are available to a diverse range of people, regardless of background. Already, there exist disparities in smartphone access. Only 66% of those without a high school education own smartphones, for example, a significant decrease from the rate of smartphone ownership among those with at least some college education (85%) and college graduates (91%)^21^. While digital health holds great potential, a commitment to justice involves ensuring that new innovations and tools do not discriminate against those who may not be as digitally informed or smartphone literate (Figs 2–5).

Although many evaluation frameworks consider ease of use or usability, access is more fundamental and the limiting factor for many seeking to use apps. Thus, in the spirit of justice, the primary level of the pyramid addresses background information and access before focusing on other related domains like usability. The components of access are multifaceted and include questions pertaining to operating system (as some apps function only on iOS or Android and many older smartphone are not able to run newer apps), cost (as price is a major barrier to use and reason for app abandonment), and offline functionality (to enable users to engage even without wifi). Offline access is important to consider as many of the most vulnerable patients are also those with the least access to internet: 29% of individuals with less than a high school education do not use the internet, compared to just 2% of college-educated adults^22^. Other questions include information about the developer and the last update, which may help indicate the presence of bugs that hamper app function and can even induce harm. For example, an analysis of app features and app quality found that days since last update was higher correlated with rating of app quality: apps that had gone more than 180 days since last being updated scored significantly lower on a quality assessment^20^. Background and access thus constitute the foundational level of the framework, since if an individual is unable to access the app and its features, the app itself is not usable and the evaluation need not proceed.

### Data safety and privacy

The second level of the framework is grounded in non-maleficence, the principle that the app should not harm individuals using it or others in society. The expectation of confidentiality is paramount in healthcare—and especially in mental healthcare, where treatment involves the disclosure of sensitive experiences. However, among existing evaluation frameworks, considerations of privacy and security feature far less prevalently than questions about short term usability^15^. While usability is often what apps market to attracts users, studies have indicated that individuals with mental illness are often deterred from using apps by concerns about the app’s ability to manage sensitive information about their treatment^23^. 70% of adults say personal data is less secure than it was 5 years ago, and 81% of Americans feel that the potential risks of data collection by companies outweigh the benefits^24^. FDA guidelines for mobile medical apps are explicit and thorough in addressing the issue of privacy^6^; many health apps, however, are exempt from these guidelines as they claim to be wellness tools. Under this guise, they often neglect to provide transparent privacy policies, despite the significant user wariness. A 2015 analysis of apps for bipolar disorder found that only 22% of surveyed apps provided a privacy policy^25^. One study revealed that top rated smoking and depression apps do not follow their own privacy policies in sharing of data with Google and Facebook despite promising not to^26^. While app store stipulations regarding privacy policies have become more stringent since 2015, it is critical to consider what data apps have access to and how personal information may be shared. This issue of data sharing has come under increasing scrutiny from the media, with the *New York Times* demonstrating in December of 2019 that apps are surreptitiously using data to continuously track precise locations^27^. Clearly, the lack of oversight for privacy and data use can have serious consequences, especially for already vulnerable populations. It is thus important to consider data use and privacy through the lens of non-maleficence.

While other app evaluation frameworks attempt to evaluate some features of security, there is ultimately high discordance (Fleiss Exact Kappa score of just 0.147) when it comes to assessing privacy and data use^10^. Furthermore, these frameworks may not be regularly updated, complicating the effort to provide an up-to-date assessment of privacy in a field that is rapidly changing.

In the APA framework, questions range from the basic “is there a transparent privacy policy that is clear and accessible before use” to “can users opt out of data collection or delete data”. While the presence of a privacy policy is a first step, it does not necessarily guarantee security. Our framework thus attempts to encourage thorough scrutiny of the policy to ensure that data is securely maintained. Users can refer to the privacy questions as they see fit, with a simple consideration of presence of a privacy policy or a more in depth assessment of issues like specific data use and third party vendors. In addition, the questions are structured so as to be responsive to changes in privacy that may arise, enabling the database to provide up to date and accurate information. While these questions cannot replace a technical review or identify apps that practice deception, they do offer a practical and feasible tool to help make better decisions around finding safe apps.

### App effectiveness and clinical foundation

The third level of the framework rests upon the principle of beneficence and is concerned with whether the app offers evidence of benefit—or at least intent of doing good for the users involved. Robust evidence of efficacy is the standard when it comes to prescribing medication or therapies. It follows that, if apps are to be successfully integrated into treatment, they too must present a strong clinical foundation. The overarching question of this level is “does the app do what it claims to do?” An app purporting to provide CBT should feature content aligning with the components of CBT and ideally evidence that those principles still translate into an effective intervention when delivered via that app.

In the current mental health app space, most claims that exaggerate benefit go unchecked and unsubstantiated. One analysis found that although 64% of the 73 reviewed apps claimed to be effective at diagnosing a mental health condition or improving symptoms, only 14% referenced design by people with lived experience, and just one app included a citation to published literature^28^. Even apps that purport to be backed by randomized controlled trials may not have a robust clinical foundation as the control groups these apps are randomized to are often inappropriate, comprising a passive control group that makes it difficult to parse whether any change was actually due to the intervention. Thus, the presence of a RCT supporting an app does not necessarily serve as a proxy for quality. A meta-analysis of standalone mental health apps investigated published literature on randomized controlled trials of mental health apps and found such small effect sizes that the authors could not recommend standalone psychological interventions at all^29^. Most concerning, a study of 69 apps for depression found six apps, downloaded more than two million times, provided inaccurate or non-existent suicide crisis helpline phone numbers^30^—underscoring the importance of ensuring apps actually do what they claim as a simple but critical bar for evaluation. These examples reveal that beneficence is not necessarily the norm when it comes to claims of app effectiveness, underscoring the need for a thorough, comprehensive system for evaluation.

While other app evaluation frameworks are concerned with credibility^15^, a focus on beneficence demands a more rigorous analysis. It is not enough for an app to make a claim backed by a vague reference to science, nor is it sufficient to accept links or phone numbers provided as evidence of credibility. The links and references should be analyzed the ensure the app strives for net benefit and does not misrepresent facts. In addition, an assessment of clinical foundation should consider both that apps appearing to be effective in research contexts may perform differently in the real world and evidence of app effectiveness may be inflated by the digital placebo effect, by which users report improvements in symptoms when using any digital product, regardless of whether the piece of technology in use is a digital intervention or merely a control^31^. Overall, studies with an active control group involving a digital control may better represent actual app effectiveness; however, given the various concerns, a framework should encourage critical assessment of any claim of effectiveness.

With beneficence in mind, the framework at this level poses questions about the app’s alignment with its claims, as well as evidence of specific benefit from academic institutions, publications, end user feedback, or research studies. Recognizing that the life cycle of an app may outpace that of published research, the framework also poses the question about attempts at feasibility and efficacy studies, with feasibility study defined as an analysis of practicality of app intervention, and efficacy study defined as a randomized controlled trial of effectiveness. Even small studies with published in smaller journals help indicate that an app is interested in developing a clinical foundation. While journal impact factor is not itself related to app evidence, it does provide an objective metric around evidence that may matter to some. Ultimately, analysis of an app at this level should identify whether an app has the intent to offer benefit for the user, and if this intent is manifested in a robust clinical foundation.

### User experience and engagement

The fourth level is grounded in the principle of autonomy, requiring that a person is able to take an active role in their care and make decisions free from coaxing and coercion.

The efficacy of any given mental health app hinges greatly upon its ability to engage a user just as current treatments for mental illness, including therapy and medication, depend on sustained use. Across mental health apps, however, low adherence and high attrition rates make it difficult to assess impact. Users engage with mental health apps for an average of less than a month, and among studies of mental health app efficacy, none have assessed long-term impact beyond the duration of the intervention. One recent study suggested that only 4% of mental health apps downloaded are used more than a single week^32^. As user preferences drive use patterns and adherence across psychological interventions^33^, our framework poses questions regarding the various features and engagement styles.

Other frameworks treat the issue of usability by asking about “ease of use”. Such a subjective metric is inherently biased and fails to account for the diversity of user preferences that drive use. We have included some of the traditional “ease of use” metrics, such as offline usability and functionality with accessibility features, as part of the first level, since they constitute components of access. Questions at level four of our framework focus on the presence or absence of different features and engagement styles that people may seek in an app, preserving autonomy and placing individual preference at the forefront of app selection.

There are numerous different engagement styles, from gamification (points and badges) to discussion boards to symptom tracking. The efficacy of each of the various engagement modalities has been supported in previous literature. Several studies, for example, bolster the potential of gaming to augment cognitive capacity in both children and adults with schizophrenia^34^. Chatbots and voice agents have become increasingly empathetic and are for some users are able to offer some of the benefits of peer support from a small handheld device^35^. With so many validated styles, determining usability is tied to personal preference. In an exploration of natural patterns of app use among primary care patients with depressive symptoms, one study identified four distinct patterns of app use: skill acquisition, social connectedness, inquisitive trial, and safety netting^36^. Focus groups have highlighted that a single approach cannot appeal to everyone; preferences in app features vary according to age and symptom severity^37^. In addition, patients are inclined to use apps which allow them to focus on their more immediate needs, as opposed to an array of features that do not facilitate their priority objectives^38^. With these findings in mind, the main questions of this level ask about the engagement style, available features, and alignment of the app and its features with user needs and priorities. The framework thus provides an objective set of considerations that respect individual autonomy in choosing an app with desired features, facilitating customization of the database to apps that suit their needs.

### Data integration towards therapeutic alliance

The final level of the framework is grounded in the principle of shared decision making. In today’s landscape, apps can fragment care, distancing an individual from their provider by segmenting different components of treatment and isolating data. Apps now provide the opportunity to access treatment modalities, such as CBT, completely removed from a medical context. This level constitutes the top layer of the pyramid because not all apps are necessarily intended to interface with the health system; some serve primarily as self-management tools. While standalone apps may boast desired features, however, apps for depression and anxiety have been shown to be two times more effective when used in conjunction with a clinician^39^. With the evident benefits of shared decision making with a clinician in mind, our framework suggests that an app intended to be used as a component of treatment in conjunction with healthcare system should allow for integration with the electronic medical record (EMR) and connection with provider or clinician. Other questions at this level pertain to the capacity for data sharing (with a clinician, peer, or social network) and the incorporation of other digital tools, like FitBit and Apple Health, that may help to augment the therapeutic alliance between an individual and their provider, optimizing shared decision making for wellness.

## Assessing reliability

App evaluators include psychologists, health professionals, academics, and end users: any interested individual can undergo the comprehensive training process to become a rater. The rating process involves a comprehensive analysis of both app store information and app functionality, requiring evaluators to download and engage with the app. App raters undergo a three hour training that involves an online information module and a practice rating of two apps, from which initial reliability is calculated. Only potential raters who exceed a kappa score of 0.7 with the reference rating are accepted as raters.

Initial testing suggests high concordance among raters for each question based upon the kappa statistic^40^. Before training, two researchers evaluated the 27 apps that appear in an iOS app store search for “schizophrenia”. Of the 80 binary questions, 72 had a Kappa score of .4 or above, indicating that 90% of the questions had at least moderate agreement despite minimal training. 63 of the 80 questions had a Kappa score above .6, demonstrating substantial or perfect agreement for 79% of the database binary questions. The inter-rater reliability improved after adding clarifying explanations for each question. When two researchers evaluated the top 29 apps appearing in an iOS app store search for “psychosis”, the average Kappa score for each level of the APA model exceeded 0.75 (Table 1). The results of these preliminary tests are currently being used to inform clarifying explanations for each question in the database, facilitating consistent crowd-sourcing.

**Table 1:** The average interrater reliability at each level of the APA model.

Framework level	Average Kappa inter-rater reliability score
Background and access	0.876
Privacy and security	0.856
Clinical foundation and app evidence	0.755
User experience: inputs and outputs	0.909
User experience: features and engagement	0.928
Data integration	0.915

The full dataset is available upon request.

The data from the first fifteen approved raters suggests that the current three hour training is sufficient to achieve a high level of reliability. This initial group comprised students and psychologists. All of the participants passed the necessary benchmark (kappa inter-rater reliability score exceeding 0.7) on their initial two practice apps. The average agreement between the raters’ evaluations and the reference answers was 0.901, while the average kappa statistic was 0.747, suggesting excellent reliability.

Comparison with current standards for app use and functionality indicates that the questions of this database are robust and flexible enough to cover nearly all use cases. A recent exploration of the characteristics, functionality, and ethical concerns of top apps for depression evaluated functionality across three different categories of use—screening, tracking and intervention—that correspond closely with our proposed questions covering various app features^41^. The NICE guidelines propose recommendations for using digital and mobile health interventions among European health systems^42^. In the latest draft of these guidelines, the recommendations for healthcare professionals in section 1.3 are all covered by questions in the database. The close alignment of these database questions with evaluation frameworks in the existing literature suggests widespread utility.

## Conclusion

This framework introduces a set of strict and objective evaluative criteria—like questions confirming the presence of a privacy policy—while leaving room for customization in line with the individual user’s needs and priorities. Different populations, such as adolescents and older adults, will have different needs in an app; the flexibility of this framework allows clinicians and providers to tailor app recommendations to these specific needs. In order to deliver effective quality care when health data is being exchanged electronically, establishing e-health literacy among users, providers, and caregivers is crucial^43^. The published database will thus include both informational and training modules to accompany the display of evaluated apps and can be accessed at apps.digitalpsych.org.

The database is enriched by widespread participation; the ultimate goal is to crowdsource evaluations such that apps can be reviewed regularly and widely. With the theoretical grounding in medical ethics, there is flexibility to amend the questions to better serve these principles as the app space continues to grow and change. What this new framework does not do is identify a “top” or “best app”; instead, it clarifies the range of options and supports them with concrete and up to date data, preserving the ability to customize the framework to individual needs. Ultimately, the database provides a public and interactive approach to data collection to create transparency, generate discussion, and provide individuals and their clinicians with the information to make the best choice for clinically meaningful app use.

## Supplementary information

Supplementary Information
