# Supplementary material for: Actionable health app evaluation: translating expert frameworks into objective metrics
Source: NPJ Digit Med. 2020 Jul 30;3:100. doi: 10.1038/s41746-020-00312-4 (PMC7393366; doi:10.1038/s41746-020-00312-4)
Supplement: Supplementary file 1 — Supplementary Information [file 41746_2020_312_MOESM1_ESM.pdf]

Supplementary Note 1: APA Framework Questions. The questions at this link: https://www.psychiatry.org/psychiatrists/practice/mental-health-apps/app-evaluation-model were developed in concert with the American Psychiatric Association's Expert App Evaluation Panel. They have been operationalized to inform the database.

Supplementary Table 1: Database Questions with clarifying descriptions for each. The level of the APA framework to which each question corresponds is included, depicting how the operationalized questions relate to the APA framework.

Question	Corresponding Level	Description
App Origin		
Does it come from the government?	Background & Access	Does the app come from a government agency? Examples include apps published by the Department of Defense and Veterans Administration.
Does it come from a for-profit company or developer?	Background & Access	Most apps on the app store come from a for-profit company or individual developer. If the name of the developer is followed by "LLC" assume it is for-profit.
Does it come from a non-profit company?	Background & Access	To determine if the app developer is a non-profit company, it may be helpful to refer to the company's website. An example of a non-profit developer is the Schizophrenia and Related Disorders Association of America, which developed the Schizophrenia Health Storylines App.
Does it come from a trusted healthcare company?	Background & Access	A hospital is an example of a healthcare developer. Some apps may be developed by a university-affiliated hospital, in which case the app comes from both a healthcare company and academic institution.
Does it come from an academic institution?	Background & Access	Apps that are developed by academic institutions include apps affiliated with universities (Harvard, Stanford, Dartmouth, UCSF NYU are just a few examples of academic institutions that have developed mental health apps).
App Functionality		
Does it work on Apple(iOS)?	Background & Access	Does the app have an entry on the iOS app store?
What is the Apple version?	Background & Access	(from Apple store link)
What is the oldest iOS version supported?	Background & Access	(from Apple store link)
What was the Apple release date?	Background & Access	(from Apple store link)

When was the last Apple (IOS) update?	Background & Access	(from Apple store link)
Has the apple version been updated in the last 180 days?	Background & Access	(from Apple store link)
Number of reviews on Apple store?	Background & Access	(from Apple store link)
Rating (number of stars) on Apple store?	Background & Access	(from Apple store link)
App size on iOS?	Background & Access	(from Apple store link)
Does it work on Android?	Background & Access	Does the app have an entry on the Google Play Store?
What is the Android version?	Background & Access	(from Google Play store link)
What is the oldest Android version supported?	Background & Access	(from Google Play store link)
What was the Google play store release date?	Background & Access	(from Google Play store link)
When was the last Android update?	Background & Access	(from Google Play store link)
Has the android version been updated in the last 180 days?	Background & Access	(from Google Play store link)
Number of reviews on google play store?	Background & Access	(from Google Play store link)
Rating (number of stars) on google play store?	Background & Access	(from Google Play store link)
App size on android?	Background & Access	(from Google Play store link)
Does the app work offline?	Background & Access	Does the app work in airplane mode?
Does it have at least one accessibility feature (like adjust text size, text to voice, or colorblind color scheme adjuster)?	Background & Access	Does the app work with adjustable text size setting of the phone? Or text to voice features? Does the app have an internal setting for incresing text size?
Does it work with Spanish?	Background & Access	This information is available on the app store.
Does it work with a language other than English or Spanish?	Background & Access	This information is available on the app store.
Is the app totally free?	Background & Access	An app is totally free if there are no costs up front and no in-app purchases.
What is the cost up front?	Background & Access	Some apps may be free up front but have in-app purchases.
Are there in-app purchases?	Background & Access	Are there any functionalities of the app that are inaccessible without payment?
Is it a subscription (recurrent/monthly/annual)?	Background & Access	Does full use of the app depend upon a subscription?
Inputs & Outputs		
Input: surveys?	Background & Access	Does the app enable a user to enter surveys such as mood or symptom surveys?
Input: Diary?	Background & Access	Does the app have a journaling, diary, or free writing feature?
Input: Geolocation?	Background & Access	Does the app enable location services from the phone?

Input: contact list?	Background & Access	Can a user connect their contact list to the app?
Input: Camera?	Background & Access	Do any features of the app utilize camera input? So profile picture? Or photo diary features? Or video chat?
Input: Microphone?	Background & Access	Does the app allow a user to record using the phone microphone?
Input: step count?	Background & Access	Does the app utilize step tracking?
Input: external devices (e.g. a wearable sending direct data)?	Background & Access	Does the app connect with an external device such as a smart watch or heart rate monitor?
Input: social network?	Background & Access	Connection to social media. Does the app allow you to input social media information? For example, do you connect it to your facebook to log in? Or do you connect with social media contacts through the app?
Output: notifications?	Background & Access	Does the app send notifications? These notifications could be incoming messages, reminders from the app, or alerts.
Output: psychoeducational references/information?	Background & Access	Does the app provide psychoeducational references or information? (Note: this means the exact same thing as the question about features: psychoeducation).
Output: social network?	Background & Access	Can you post information from the app to social media? Does the app connect to social media for posting purposes?
Output: reminders?	Background & Access	Does the app allow you to set reminders? (Oftentimes these reminders will then generate notifications)
Output: graphs of data?	Background & Access	Does the app allow a user to see graphically depicted data?
Output: summary of data (in text or numbers)?	Background & Access	Does the app provide written summaries of data (description of data apart from a graph)?
Output: link to formal care/coaching?	Background & Access	Does the app connect a user with a healthcare provider? A licensed therapist or clinician?
Privacy & Security		
Is there a privacy policy?	Data Safety & Privacy	The following questions involve reading the privacy policy, which will be linked to the app store if the app has one.
Does the app declare data use and purpose?	Data Safety & Privacy	What information is the app collecting and for what purpose?
Does the app report security measures in place?	Data Safety & Privacy	How is data protected? Does the app claim to collect and share data securely?
Is PHI shared?	Data Safety & Privacy	PHI refers to personal health information that is entered into the app (name, birthday, content of messages, mental health information). Data is shared if it leaves the app in any way.
Is de-identified data shared?	Data Safety & Privacy	De-identified data is information that has been stripped of personally identifiable attributes.
Is anonymized/aggregate data shared?	Data Safety & Privacy	Most apps collect and share aggregate use data. This anonymized data has no traceable link to an individual.
Can you opt out of data collection?	Data Safety & Privacy	Is there a way for a user to indicate that they don’t want to app to collect or share their data?

Can you delete your data?	Data Safety & Privacy	Can user data be deleted? Some apps may retain data permanently.
Is the user data stored only on the device?	Data Safety & Privacy	User data is either stored locally (exclusively on the device) or on a server.
Is the user data stored on a server?	Data Safety & Privacy	If the data is stored only on the device, then it won't be stored on a server.
Does the app have a crisis management feature?	Data Safety & Privacy	The presence of a crisis management feature is often indicated in the privacy policy. A crisis management feature refers to an app's emergency response: does the app provide a hotline number that can be called if user input suggests a crisis?
Does the app claim it meets HIPAA?	Data Safety & Privacy	Does the app specify it is compliant with HIPAA and that health information is protected compliant with HIPAA standards?
Reading level of the privacy policy (what grade reading level)?	Data Safety & Privacy	Flesch-Kincaid reading grade level: https://readabilityformulas.com/free-readability-formula-tests.php (just copy and paste privacy policy in).
Does the app use 3rd party vendors (i.e. google analytics, etc)?	Data Safety & Privacy	Does the app mention use of third parties?
Evidence & Clinical Foundation		
Does the app appear to do what it claims to do?	App Effectiveness and Clinical Foundation	If the app claims to offer CBT, for example, is there evidence that CBT is provided on the app?
Is the app patient facing?	App Effectiveness and Clinical Foundation	This will depend on the terms of the search. Is the app relevant for an individual with the condition specified in the search? Is it intended for patient use (some apps, for example, may be diagnosis guides and intended for use by healthcare providers but not patients).
How many feasibility/usability studies?	App Effectiveness and Clinical Foundation	How many studies about the feasibility of this specific app's use have been published?
What is the highest feasibility impact factor?	App Effectiveness and Clinical Foundation	What is the impact factor of the journal in which the feasability study is published? This can easily be found with a quick google search. If it's not immediately evident, put "0" (assume the journal doesn't have an impact factor.
How many evidence/efficacy studies?	App Effectiveness and Clinical Foundation	How many studies about the efficacy of this app to accomplish what it claims to do have been published?
What is the highest efficacy impact factor?	App Effectiveness and Clinical Foundation	What is the impact factor of the journal in which the efficacy study is published? This can easiliy be found with a quick google search. If it's not immediately evident, put "0" (assume the journal doesn't have an impact factor.
Can the app cause harm?	App Effectiveness and Clinical Foundation	Does the app make recommendations or suggestions that directly defy clinical guidelines? Does it include overtly false information, like a

		suicide hotline number that doesn't actually work?
Does the app provide any warning for use?	App Effectiveness and Clinical Foundation	Is there any warning for a user that the app is not intended to replace medical care?
Features & Engagement Style		
Features: mood tracking?	User Experience & Engagement	Does the app provide surveys where a user can enter their mood data?
Features: medication tracking?	User Experience & Engagement	Designated medication tracking feature?
Features: sleep tracking?	User Experience & Engagement	Does the app track sleep, either in conjunction with a wearable or through user-entered information?
Features: physical exercise tracking?	User Experience & Engagement	Does it allow a user to track duration or content of physical exercise?
Features: psychoeducation?	User Experience & Engagement	Does it provide definitions, explanations of different diagnoses? Is it didactic?
Features: journaling?	User Experience & Engagement	Is there a place for the user to journal or free write?
Features: picture gallery/hope board?	User Experience & Engagement	Does the app allow a user to curate a gallery of saved and searched images and quotes?
Features: mindfulness?	User Experience & Engagement	Any mindfulness exercises? May include deep breathing but not necessarily.
Features: deep breathing?	User Experience & Engagement	Does the app offer exercises in deep breathing?
Features: iCBT or sleep therapy?	User Experience & Engagement	Doe the app offer sleep therapy of any kind (including iCBT, a targeted sleep intervention)?
Features: CBT?	User Experience & Engagement	Does the app provide cognitive-behavioral therapy?
Features: ACT?	User Experience & Engagement	Does the app provide Acceptance and Commitment Therapy?
Features: DBT?	User Experience & Engagement	Does the app provide dialectical behavior therapy?
Features: peer support?	User Experience & Engagement	Does the app offer connection to peer specialists or individuals with lived experience?
Features: connection to coach/therapist?	User Experience & Engagement	The app has a built-in way to connect with a provider or coach.

Features: biodata?	User Experience & Engagement	Does the app collect heart rate or skin conductance?
Features: goal setting/habits?	User Experience & Engagement	Productivity feature allowing user to set and check in on goals.
Features: physical health exercises?	User Experience & Engagement	Something like 7 minute workout that actually gives a workout (this is a recommendation of exercises, NOT tracking.
Features: Bbot interaction (like with virtual character)?	User Experience & Engagement	The app allows a user to interact with a virtual character.
Features: Biofeedback with sense data (eeg, HRV, skin conductance, etc)?	User Experience & Engagement	The app uses biodata to provide feedback/recommendations (an app that will recommend more breathing exercises to respond to high heart rate, for example).
Engagement style: user generated data?	User Experience & Engagement	Does the user engage with the app by inputting their own data (for example, mood tracking or diary)?
Engagement style: chat/message based?	User Experience & Engagement	User can send and receive messages.
Engagement style: is it a screener/assessment?	User Experience & Engagement	Examples include PHQ9, GAS7, etc.
Engagement style: real time response?	User Experience & Engagement	Someone will reply to your chat right away.
Engagement style: Asynchronous response?	User Experience & Engagement	There are no immediate responses to chats; responses come at predetermined intervals (once a day; every four hours; etc)
Engagement style: gamification (points, badges)?	User Experience & Engagement	User can win points and prizes for engaging with the app.
Engagement style: videos?	User Experience & Engagement	App includes videos user can view.
Engagement style: audio/music/scripts?	User Experience & Engagement	Does the app provide music or audio experiences? Some meditation apps, for example, utilize audio sessions.
Engagement style: AI support?	User Experience & Engagement	Interaction is not with a real person but with a bot.
Engagement style: peer support?	User Experience & Engagement	Peer is defined as a person with lived experience and support involves actually communcating (so not just watching a video).
Engagement style: network support?	User Experience & Engagement	Network is defined as someone (like family or friend) who is actually known.by the user outside the app. An example is an app that allows a user to communicate with family members about relevant health information.
Engagement style: Collaborative with provider/other?	User Experience & Engagement	Does it allow for direct collaboration with a provider or clinician? Beyond just being able to share your data.
App Use		

Is it a self-help/self-management tool?	Data Integration towards Therapeutic Alliance	Provides activities that can be used for self-help and self-management, such as mood or symptom tracking or mindfulness exercises.
Is it a reference app?	Data Integration towards Therapeutic Alliance	Provides information and references but not necessarily activities. psychoeducation first.
Is it intended for hybrid use with a clinician in conjunction with treatment plan?	Data Integration towards Therapeutic Alliance	Is the app intended to be used as an adjunct to care? Apps that have built-in methods of connecting with a provider meet this criteria. However, a teletherapy app would not be intended for hybrid care, as the app replaces in-person care.
Interoperability & Data Sharing		
Do you own your data?	Data Integration towards Therapeutic Alliance	This can be found in the privacy policy of the app but is relevant for data sharing capacity. Can a user see and access their data from the app?
Can you email or export your data?	Data Integration towards Therapeutic Alliance	Can data be downloaded or exported, or emailed straight from the app?
Can you send your data to a medical record?	Data Integration towards Therapeutic Alliance	Does the app sync with EMR? (most apps are not currently equipped with connections to medical record, so this will likely be no)
